# High-Efficiency Sky Blue-To-Green Fluorescent Emitters Based on 3-Pyridinecarbonitrile Derivatives

**DOI:** 10.3389/fchem.2019.00254

**Published:** 2019-04-24

**Authors:** Yuki Masuda, Hisahiro Sasabe, Hiroki Arai, Natsuki Onuma, Junji Kido

**Affiliations:** ^1^Department of Organic Materials Science, Graduate School of Organic Materials Science, Yamagata University, Yamagata, Japan; ^2^Frontier Center for Organic Materials (FROM) Yamagata University, Yamagata, Japan; ^3^Research Center for Organic Electronics (ROEL), Yamagata University, Yamagata, Japan

**Keywords:** solid-state emission, organic light-emitting device, donor–acceptor system, thermally activated delayed fluorescence, photochemistry

## Abstract

The pyridinecarbonitrile derivative is well known as an acceptor unit in fluorescent materials. However, its use in thermally activated delayed fluorescent (TADF) emitters is very limited compared with its benzenecarbonitrile counterparts. Very recently, we developed a series of 4-pyridinecarbonitrile, so-called isonicotinonitrile derivatives, as a highly efficient sky blue-to-green TADF emitters realizing low-drive-voltage organic light-emitting devices (OLEDs). In this work, we contributed new design and development for three 3-pyridinecarbonitrile-based TADF emitters named **2AcNN**, **2PXZNN**, and **5PXZNN**. Among these emitters, a sky blue emitter, **2AcNN**, showed a maximum external quantum efficiency (*η*_ext,max_) of 12% with CIE (0.19, 0.36). While green emitters, **5PXZNN** and **2PXZNN**, realized highly efficient TADF OLEDs with a *η*_ext,max_ of 16–20%. Introduction of electron-donor moiety into the 2-position of 3-pyridinecarbonitrile contributes a larger overlapping of frontier molecular orbitals (FMOs) and stronger intramolecular charge transfer (ICT) interaction generating efficient TADF emitters.

## Introduction

High-efficiency organic light-emitting devices (OLEDs) have attracted significant attention due to their ability to yield energy savings in small- to large-area flat-panel displays and general lighting applications (Walzer et al., 2007; Sasabe and Kido, 2013; Adachi, 2014; Im et al., 2017; Wong and Z.-Colman, 2017; Yang et al., 2017; Kim and Kim, 2018; Komatsu et al., 2018). Organic phosphorescent and thermally activated delayed fluorescent (TADF) emitters can convert all electrogenerated molecular excitons such as singlets and triplets to photons achieving an electron-to-photon conversion efficiency of up to 100%. Recently, the development of pure organic TADF emitters has been focused on simultaneously realizing cost-effective and high-performance OLEDs compared with its phosphorescent counterparts (Uoyama et al., 2012; Adachi, 2014; Kaji et al., 2015; Lin et al., 2016; Seino et al., 2016; Im et al., 2017; Liu et al., 2017; Rajamalli et al., 2017; Wong and Z.-Colman, 2017; Yang et al., 2017; dos Santo et al., 2018; Komatsu et al., 2018; Sasabe et al., 2018; Wu et al., 2018). In principle, TADF emitters consist of electron-donor (D) and electron-acceptor (A) moieties realizing efficient intramolecular charge transfer (ICT). The connection between D and A moieties is generally accompanied with a small overlap in the frontier molecular orbital (FMO) between the highest occupied molecular orbital (HOMO) and the lowest unoccupied molecular orbital (LUMO), in other words, a small energy difference between singlet and triplet energies (Δ*E*_ST_). Among the A units, cyano-containing aromatic moieties, such as benzenecarbonitriles and pyridinecarbonitriles, are very effective in developing high-performance TADF emitters. However, compared with well-known benzenecarbonitrile derivatives, more electron-deficient pyridinecarbonitrile-based counterparts have been relatively unexplored.

In 2015, Liu et al. reported a pyridinedicarbonitrile/carbazole-conjugated molecule, namely **CPC**, in which pyridinedicarbonitrile and carbazole units are directly linked, showing a photoluminescent quantum yield (*η*_PL_) of 49.7% in host doped film (Liu et al., 2015). The **CPC** showed efficient sky blue emission with a maximum external quantum efficiency (*η*_ext,max_) of 21% and Commission Internationale de l'Éclairage (CIE) coordinates (0.20, 0.35). This is the first report to use pyridinecarbonitrile as an acceptor moiety of a TADF emitter. In 2016, Pan and co-workers developed several pyridinedicarbonitrile/dimethylacridine-conjugated molecules (Pan et al., 2016). Among these molecules, **Py2** and **Py5** showed a high *η*_PL_ of 89–92% and yielded efficient greenish blue OLED with *η*_ext,max_ at 23–24%. CIE coordinates were (0.24, 0.49) for **Py2** and (0.28, 0.54) for **Py5**. In 2017, Sasabe et al. developed a series of 4-pyridinecarbonitrile, so-called isonicotinonitrile derivatives, as highly efficient sky blue-to-green TADF emitters realizing low-drive-voltage OLEDs (Sasabe et al., 2017). Sky blue emitter **26AcINN** exhibited a low turn-on voltage of 2.9 V, a maximum power efficiency (*η*_p,max_) of 66 lm W^−1^, and a *η*_ext,max_ of 22% with CIE coordinates (0.22, 0.45). Meanwhile, green emitter **26PXZINN** exhibited a low turn-on voltage of 2.2 V, a high *η*_p,max_ of 99 lm W^−1^, and a *η*_ext,max_ of 22% with CIE coordinates (0.37, 0.58). As mentioned above, although pyridinecarbonitrile-based TADF emitters exhibited very promising OLED performances, they have been relatively unexplored so far.

In this work, we focused on 3-pyridinecarbonitrile derivatives as TADF emitters aiming bluer emission based on a larger energy gap than that of the corresponding 4-pyridinecarbonitrile derivatives, and aimed to enrich the materials science of cyano-containing aromatic compounds. We designed and developed three 3-pyridinecarbonitrile-based TADF emitters labeled **2AcNN**, **2PXZNN**, and **5PXZNN**. Among these emitters, a blue emitter, **2AcNN**, exhibited a sky blue emission with CIE coordinates (0.19, 0.36), *η*_ext,max_ of 12%, and *η*_p,max_ of 28.8 lm W^−1^. Meanwhile, a green emitter, **2PXZNN**, realized a high efficiency TADF OLED with CIE coordinates (0.35, 0.55) and *η*_ext,max_ of 20.8%.

## Results and Discussion

### Density Functional Theory Calculation and Synthesis

**Figure 1:**
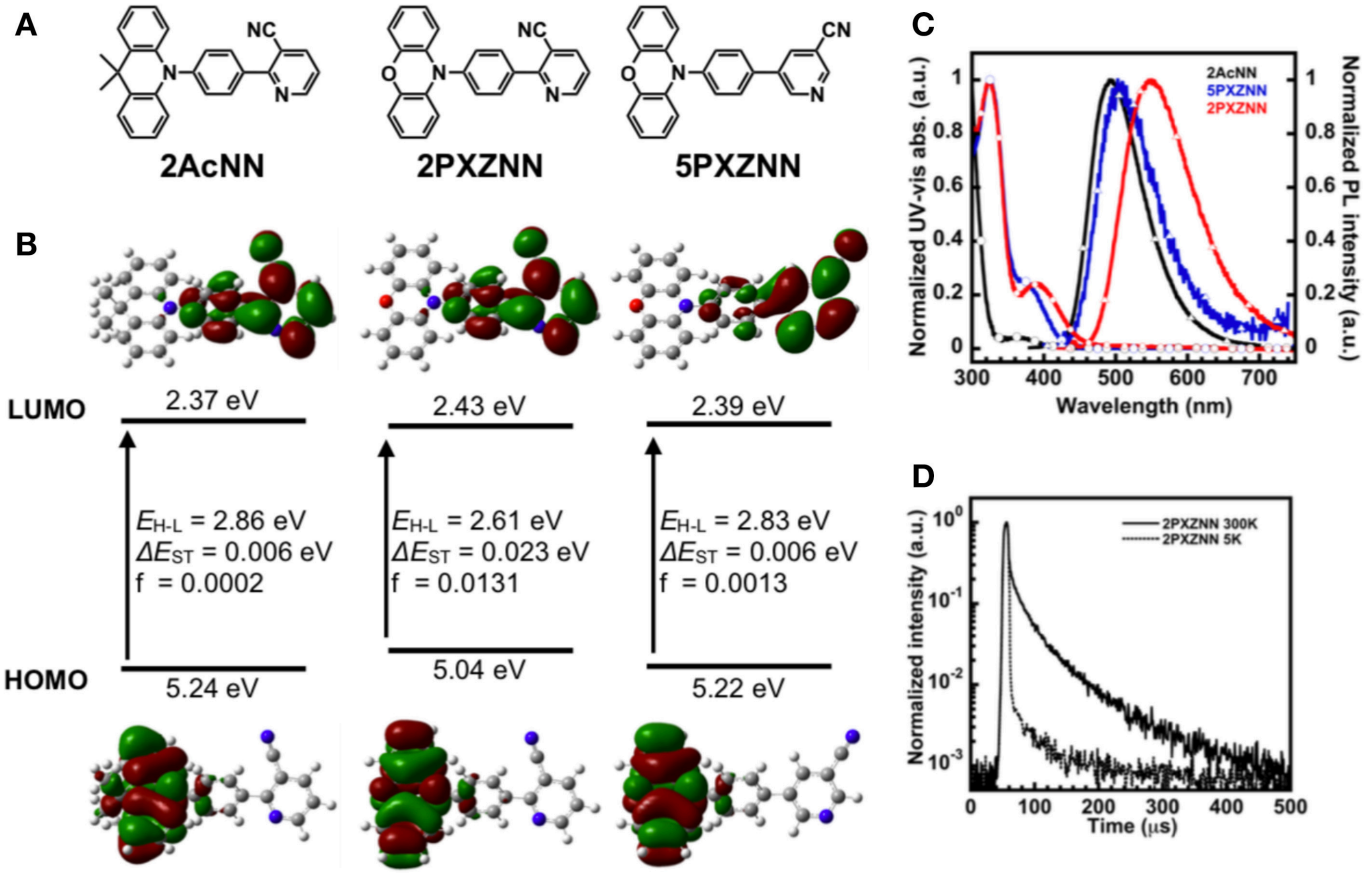
**(A)** Chemical structures. **(B)** Highest occupied molecular orbital (HOMO) and lowest unoccupied molecular orbital (LUMO) distribution, energy levels, energy differences between HOMO and LUMO (Δ*E*_H−L_), singlet and triplet excited states (Δ*E*_ST_), and oscillator strength (f). **(C)** Ultraviolet–visible (UV–vis) absorption and PL spectra of **2AcNN**, **2PXZNN**, and **5PXZNN** in a toluene solution (10^−5^ M). **(D)** Transient photoluminescent decay curves at 5 and 300 K for **2PXZNN**.

In previous work, we successfully developed a sky blue TADF emitter, **26AcINN**, with a *η*_ext,max_ of 22% (Sasabe et al., 2017). However, color coordinates were not located in the blue region. Therefore, in order to obtain bluer emission compared with the 4-pyridinecarbonitrile derivatives, we used 3-pyridinecarbonitrile with reduced π-conjugation as an A unit. In addition, to get an effective molecular design and expand the materials chemistry of the pyridinecarbonitrile derivatives, we designed three types of 3-pyridinecarbonitrile-based TADF emitters, **2AcNN**, **2PXZNN**, and **5PXZNN**, using dimethylacridine (**Ac**) and phenoxiazine (**PXZ**) donors. Prior to preparing the emitters, we conducted density functional theory (DFT) calculations to estimate the geometric structure, the energy difference between HOMO and LUMO (Δ*E*_H−L_), and the *E*_S_, *E*_T_, and Δ*E*_ST_ of **2AcNN**, **2PXZNN**, and **5PXZNN** (Figure 1A and Table S1). These derivatives exhibited small Δ*E*_ST_ (<0.02 eV) values, which is consistent with TADF emissions. The electron cloud distribution in Figure 1B shows that the HOMO was located on the donor unit, whereas the LUMO was located on the phenylpyridinecarbonitrile skeleton. Among the PXZ derivatives, **5PXZNN** showed a larger energy gap than **2PXZNN**. To verify these differences, we also conducted DFT calculations of the phenylpyridinecarbonitrile skeleton (Figure S1). As a result, **5PXZNN** showed smaller electron distributions of LUMO on the phenyl ring compared with **2PXZNN**, resulting in smaller overlapping of FMO and weaker ICT interaction causing a larger energy gap. The FMOs were almost completely separated with a small overlap. The synthetic routes of the target compounds are shown in Schemes S1–3. These compounds were readily synthesized through a Suzuki–Miyaura coupling reaction between a halogenated 3-pyridinecarbonitrile precursor and a donor-substituted 4-phenylboronate (Komatsu et al., 2016) with 67–78% yield, and multigram amounts of product were readily obtained. The target compounds were characterized using ^1^H NMR, ^13^C NMR, mass spectrometry, and elemental analysis.

### Thermal and Photophysical Properties

Products were purified through train sublimation before device fabrication. The purity of the compounds was evaluated at over 99% using high-performance liquid chromatography. The compounds can be used for further thermal and photophysical investigation, and OLED evaluation with no influence from impurities. The thermal properties of the materials were estimated via thermogravimetric analysis (TGA) and differential scanning calorimetry (DSC). The materials exhibited high thermal stability with a weight loss of 5% (*T*_d5_) at temperatures over 300°C. The glass transition temperatures (*T*_g_) were observed at 56°C for **2AcNN**, 50°C for **2PXZNN**, and 57°C for **5PXZNN**. The *T*_g_ values were relatively low due to the low molecular weight of these emitters. The physical properties of the materials were evaluated in the solid-state film. The ionization potential (*I*_p_) levels, measured via photoelectron yield spectroscopy (PYS), were observed at ~-5.7 to −5.8 eV. The optical energy gap (*E*_g_) was taken as the point of intersection of the normalized ultraviolet–visible (UV–vis) absorption spectra. The electron affinity (*E*_a_) levels were estimated at ~-3.0 eV, by subtracting the optical *E*_g_ from the *I*_p_ level.

The optical properties of the materials were evaluated under a dilute toluene solution (10^−5^ M). The UV–vis absorption and PL spectra of the pyridinecarbonitrile derivatives obtained at room temperature are on display in Figure 1C. The PL peak wavelength was observed at 492 nm for **2AcNN**, 550 nm for **2PXZNN**, and 503 nm for **5PXZNN**. As predicted from the DFT calculations, **2AcNN** showed sky blue emission and a shorter wavelength based on the weaker ICT character among these three emitters. On the other hand, **2PXZNN** showed green emission and a longer wavelength due to the stronger ICT character as shown in the UV–vis absorption spectra.

With regard to the application of OLEDs, the photophysical properties of the thin film have to be evaluated. Because a TADF emitter can harvest all the molecular excitons from singlets and triplets, the *E*_T_ of host materials should be higher than that of the emitters suppressing the triplet exciton quenching for high-efficiency OLEDs. Therefore, the PL spectra were subsequently investigated in a host matrix of bis[2-(diphenylphosphino)phenyl]ether oxide (DPEPO) with a high triplet energy (*E*_T_) of 3.30 eV and a host matrix of 4,4′-bis(carbazol-9-yl)biphenyl (CBP) with *E*_T_ of 2.60 eV. As shown in Figure S2, the emission peaks in DPEPO films doped with 10 wt% **2AcNN**, **5PXZNN**, and **2PXZNN** were located at 477, 492, and 521 nm, respectively. Furthermore, the emission peaks in CBP films doped with 10 wt% **5PXZNN** and **2PXZNN** were almost the same as those observed in the DPEPO host. *η*_PL_ differed between the DPEPO and CBP hosts. The *η*_PL_ value of **5PXZNN** was lowered in CBP (*η*_PL_ = 31%) compared with that in DPEPO (*η*_PL_ = 59%), but in the case of **2PXZNN**, it remained unchanged (*η*_PL_ values: 68% in DPEPO and 69% in CBP). These results suggested that **5PXZNN** had higher *E*_T_ than that of CBP and was quenched by CBP. The TADF character was subsequently confirmed by examining the PL decay curves of the emitter doped host films at various temperatures (i.e., 5 and 300 K; Figure 1D; Figure S3). The delayed PL intensities of the three materials increased at 300 K, thereby indicating the presence of TADF. The transient PL decay curves of the 10 wt%-doped DPEPO or CBP films exhibited double-exponential decay with delayed lifetimes (τ_d_) of 264, 175, and 53 μs for **2AcNN**, **5PXZNN**, and **2PXZNN**, respectively. The τ_d_ are relatively long compared with the well-known green TADF emitters, such as **4CzIPN** (τ_d_ = 5.1 μs; Uoyama et al., 2012), most likely because of the relatively large Δ*E*_ST_ of ~0.4 eV. Considering the combined results from the DFT calculations and photophysical experiments, an electron-donor should be introduced into the 2-position of 3-pyridinecarbonitrile to create a superior TADF emitter. The introduction of an electron-donor into the 2-position contributes a larger overlapping of FMOs and stronger ICT interaction for efficient TADF behaviors leading to shorter τ_d_. All thermal and photophysical properties of the pyridinecarbonitrile derivatives are summarized in Table 1 and Table S2.

**Table 1:** Thermal and optical properties.

**Compound**	***Mw*.**	***T*_**g**_/*T*_**m**_/*T*_**d5**_ [**°**C]^**a**^**	***I*_**p**_/*E*_**a**_/*E*_**g**_ [eV]^**b**^**	***E*_**S**_/*E*_**T**_/Δ*E_***ST***_* [eV]^**c**^**	**τ_**d**_ [μs]^**d**^**	***η*_**PL**_ [%]**
**2AcNN**	387.5	56/195/317	−5.73/−2.99/2.74	3.08/2.71/0.37	264	64^e^
**2PXZNN**	361.4	50/161/322	−5.70/−3.12/2.58	2.74/2.60/0.14	53^f^	68^e^/69^f^
**5PXZNN**	361.4	57/239/321	−5.80/−3.06/2.74	2.91/2.52/0.40	175	59^e^/31^f^

a *T_g_ and T_m_ were measured by DSC, and T_d5_ was measured by TGA*.

b *I_p_ was measured by PYS, E_g_ was taken as the point of intersection of the normalized absorption spectra, and E_a_ was calculated from I_p_ and E_g_*.

c *The onset of delayed PL of the neat film was measured using a streak camera and ΔE_ST_ = E_S_-E_T_*.

d *Delayed fluorescent lifetime of the 10 wt%-doped DPEPO film*.

e *PL quantum yield of the 10 wt%-doped DPEPO film*.

f *CBP was used instead of DPEPO*.

### Organic Light-Emitting Device Performance

**Figure 2:**
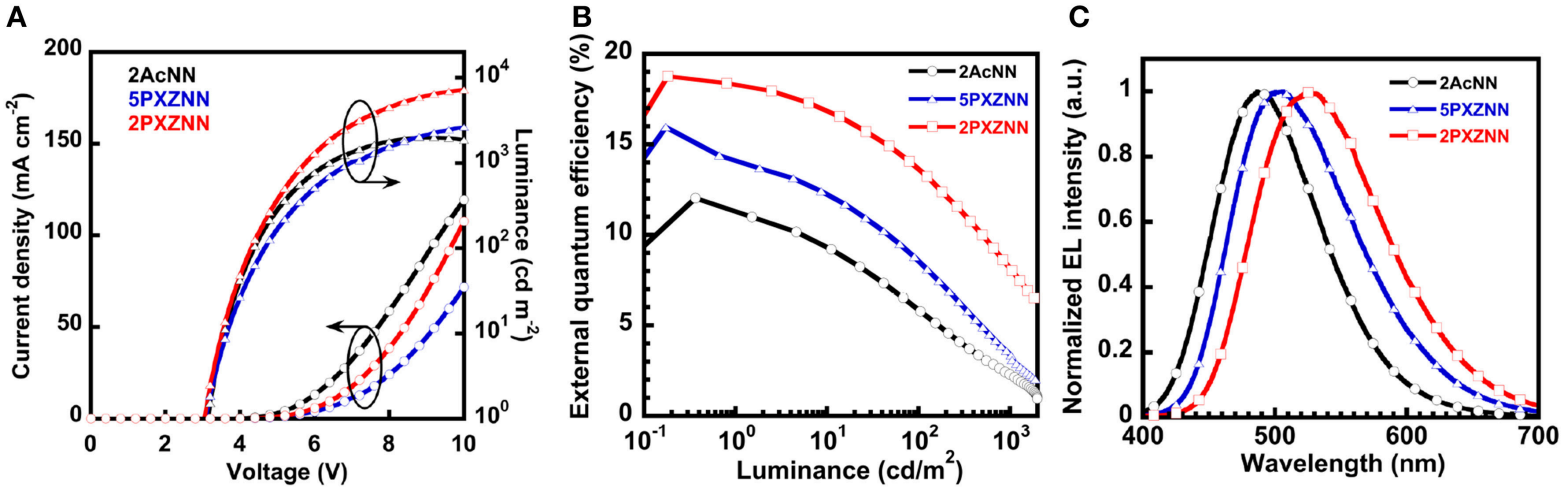
Organic light-emitting device (OLED) performances of **2AcNN**-, **5PXZNN**-, and **2PXZNN**-based devices. **(A)** *J*–*V*–*L* characteristics. **(B)** *η*_ext_-*L* characteristics. **(C)** EL spectra at 1 mA.

Next, we evaluated the OLED performance for three emitters, **2AcNN**, **5PXZNN**, and **2PXZNN**. We used a carrier- and exciton-confining device to maximize OLED performance. We employed di-[4-(*N,N*-ditolyl-amino)-phenyl]cyclohexane (TAPC) as a hole transport layer (HTL), and TAPC with a shallow *E*_a_ of −2.0 eV to effectively block electrons. 3,3″,5,5′-Tetra(3-pyridyl)-1,1′;3′1″-terphenyl (B3PyPB; Sasabe et al., 2008a,b) was used as an electron transport layer (ETL), and B3PyPB with a deep *I*_p_ of −6.6 eV effectively blocked the hole leakage. By using a combination of TAPC and B3PyPB, we were able to confine all holes and electrons in the emission layer (EML) to create superior carrier balance. In addition, *N,N*-dicarbazoyl-3,5-benzene (mCP) was inserted at the interface between HTL and EML to prevent the exciton-quenching between TAPC and emitter molecules. This is because the *η*_PL_ value of **2AcNN** was lower in TAPC (*η*_PL_ = 42%) compared with neat film of **2AcNN** (*η*_PL_ = 60%). DPEPO was used as a host material, and the *E*_T_ levels of TAPC, mCP, DPEPO, and B3PyPB were 2.98, 3.00, 3.30, and 2.77 eV, respectively. The chemical structures of the materials used in this study are shown in Figure S4. Therefore, we were able to suppress exciton quenching from the perspective of *E*_T_ confinement and maximize OLED performance. All chemical structures for the materials used in this study are shown in Figure S4. Three types of OLEDs with the structures of [ITO/triphenylamine-containing polymer: 4-isopropyl-4′-methyldiphenyl-iodonium tetrakis(pentafluorophenyl)borate (PPBI; Kido et al., 1997) (20 nm)/TAPC (25 nm)/mCP (5 nm)/10 wt% emitter-doped DPEPO (20 nm)/B3PyPB (50 nm)/LiF (0.5 nm)/Al (100 nm)] were fabricated. Figure S5A shows the energy diagrams for these devices. All peaks in the EL spectra originate from emitters with no emissions arising from neighboring materials. The current density–voltage–luminance (*J*–*V*–*L*) characteristics are shown in Figure 2A, and the external quantum efficiency–luminance (*η*_ext_-*L*) characteristics are shown in Figure 2B. OLED performance is summarized in Table 2. The EL emission peaks located at 486, 506, and 527 nm are linked to **2AcNN**, **5PXZNN**, and **2PXZNN**, respectively (Figure 2C). Among these, a blue emitter, **2AcNN**, showed a sky blue emission with CIE (0.19, 0.36), *η*_ext,max_ of 12%, and *η*_p,max_ of 28.8 lm W^−1^. The operating voltage at 1 cd m^−2^ was relatively low, at 3.1 V. By contrast, a green emitter, **2PXZNN**, realized a high-efficiency TADF OLED with CIE (0.35, 0.53) and *η*_ext,max_ of 18.8%. The operating voltage at 1 cd m^−2^ was recorded at 3.0 V and was almost identical to the **2AcNN**-based sky blue OLED. Compared with state-of-the-art green OLEDs, the operating voltage can be reduced significantly.

**Table 2:** Summary of OLED performances.

**Emitter**	***V*_**on**_ [V]^**a**^**	***V*_**100**_/*η*_**p, 100**_/*η*_**c, 100**_/*η*_**ext, 100**_ [V/lm W^**−1**^/cd A^**−1**^/%]^**b**^**	***V*_**1000**_/*η*_**p, 1000**_/*η*_**c, 1000**_/*η*_**ext, 1000**_ [V/lm W^**−1**^/cd A^**−1**^/%]^**c**^**	***η*_**p, max**_/*η*_**c, max**_/*η*_**ext, max**_ [lm W^**−1**^/cd A^**−1**^/%]^**d**^**	**CIE (x, y)^**e**^**
**2AcNN**	3.1	4.4/9.7/13.4/5.9	6.4/2.5/5.1/2.3	28.8/27.5/12.0	(0.19, 0.36)
**5PXZNN**	3.1	4.7/15.8/23.4/8.6	7.0/4.1/9.1/3.4	48.9/43.6/15.9	(0.25, 0.45)
**2PXZNN**	3.0	4.3/30.5/41.6/13.7	5.8/13.5/24.8/8.2	64.0/57.0/18.8	(0.33, 0.53)
**2PXZNN**^f^	3.0	3.6/53.7/61.5/19.3	4.4/31.8/44.0/13.8	65.2/66.4/20.8	(0.35, 0.55)
**2PXZNN**^g^	2.4	3.1/54.1/53.8/17.3	4.1/29.2/38.0/12.3	71.6/56.8/18.3	(0.37, 0.56)

a *Turn-on voltage (V) at 1 cd m^−2^*.

b *Power efficiency (*η*_p_), current efficiency (*η*_c_), voltage (V), and external quantum efficiency (*η*_ext_) at 100 cd m^−2^*.

c **η*_p_, *η*_c_, V, and *η*_ext_ at 1,000 cd m^−2^*.

d **η*_p_, *η*_c_, and *η*_ext_ at maximum*.

e *CIE at 100 cd m^−2^*.

f *Device using CBP-doped 2PXZNN*.

g *Device using TCTA-doped and CBP-doped 2PXZNN as a double emission layer and B4PyPPM*.

### Low-Operating-Voltage 2PXZNN-Based Organic Light-Emitting Devices

**Figure 3:**
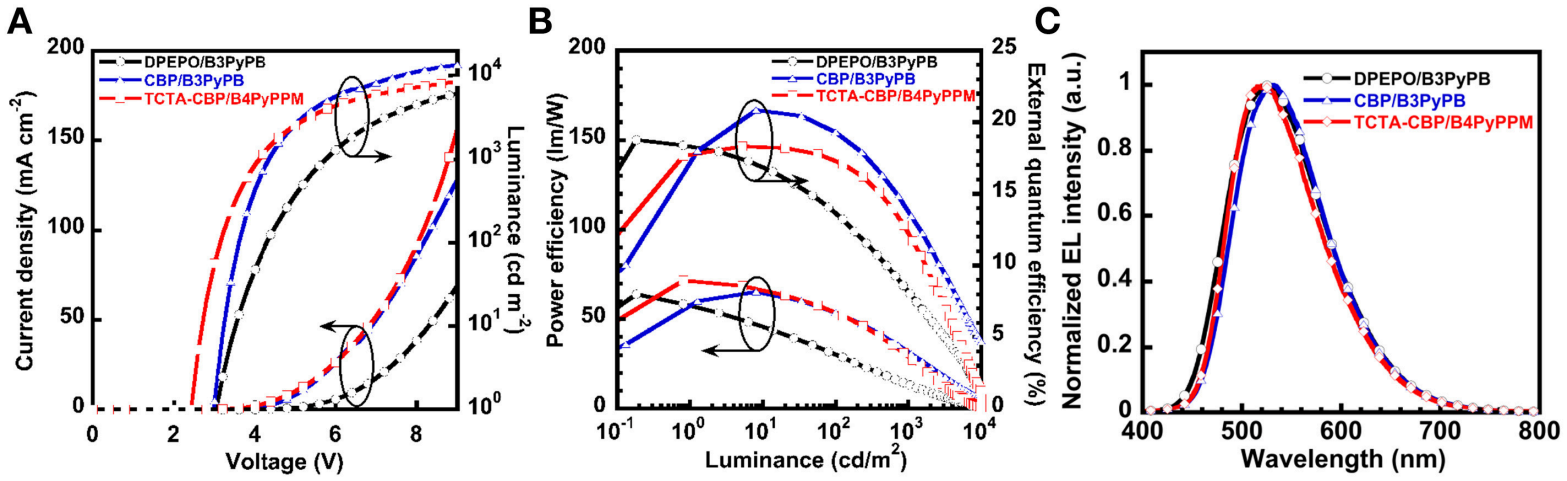
OLED performances of optimized **2PXZNN**-based devices. **(A)** *J*–*V*–*L* characteristics. **(B)** *η*_ext_-*L* characteristics. **(C)** EL spectra at 1 mA.

In the previous section, we also used DPEPO as a host material for a green emitter, **2PXZNN**. As a result, **2PXZNN**-based OLED showed a relatively high operating voltage of 3.0 V at 1 cd m^−2^. To further reduce operating voltage, we simply substituted host material DPEPO with CBP. Note that the **2PXZNN**-doped CBP film exhibited a *η*_PL_ value of 69%, similar to that of a DPEPO film. An OLED with an EML of 10 wt% **2PXZNN**-doped CBP (20 nm) was fabricated. Figure S5B shows the energy diagram. The *J*–*V*–*L* characteristics are shown in Figure 3A, and the *η*_ext_-*L*–*η*_p_ characteristics are shown in Figure 3B. The peak in the EL spectra at 532 nm originates from **2PXZNN**, with no emissions arising from neighboring materials (Figure 3C). The device showed green emission with CIE (0.35, 0.55), higher *η*_ext,max_ of 20.8%, and *η*_p,max_ of 65 lm W^−m^. Compared with a DPEPO-based device, the CBP-based device exhibited slightly higher *η*_ext,max_ value. The operating voltage was recorded at 3.0 V, similar to that of a DPEPO-based device. However, much higher current density and luminance were obtained using a CPB host. This is most likely due to the improved carrier balance in the EML. The CBP host was considered to contribute an increase in hole carrier in the EML compared with a DPEPO host. Encouraged by these results, we further improved the device structure using a double EML device using 4,4,4-tris(N-carbazolyl)triphenylamine (TCTA) with shallower *I*_p_ and *E*_a_ than CBP to increase the density of the hole carriers. Note that the *η*_PL_ value of a 10 wt% **2PXZNN**-doped TCTA film was recorded to be 64%. In addition, we used B4PyPPM as an ETL (Sasabe et al., 2008a,b, 2011). B4PyPPM has a deeper *E*_a_ than B3PyPB, enabling efficient electron injection leading to low-operating-voltages. An OLED with the structure [ITO/triphenylamine-containing polymer: PPBI (20 nm)/TAPC (30 nm)/10 wt% **2PXZNN**-doped TCTA (10 nm)/10 wt% **2PXZNN**-doped CBP (10 nm)/B4PyPPM (50 nm)/LiF (0.5 nm)/Al (100 nm)] was fabricated. Figure S5C displays the energy diagram. OLED performance is summarized in Table 2. The EL emission peak is located at 523 nm with CIE (0.37, 0.56), high *η*_ext,max_ of 18%, and *η*_p,max_ of 72 lm W^−m^. The operating voltage was recorded as 2.4 V, a reduction of 0.6 V compared with the previous device, leading to a much higher *η*_p,max_ of 71.6 lm W^−m^.

## Conclusion

In this work, we focused on relatively unexplored pyridinecarbonitrile derivatives, particularly on 3-pyridinecarbonitrile derivatives for an efficient TADF emitter aiming for bluer emission, and aimed to enrich the science of cyano-containing aromatic compounds. We developed three new types of 3-pyridinecarbonitrile-based TADF emitters, labeled **2AcNN**, **2PXZNN**, and **5PXZNN**. These compounds can be readily prepared through one-step Suzuki–Miyaura cross-coupling reaction with 67–78% yields. These emitters exhibited relatively high *η*_PLs_ of 59−69% with low Δ*E*_ST_ values of 0.14–0.40 eV in host matrices, such as DPEPO and CBP. Following photophysical investigations of the EML, three emitters appeared to achieve TADF behavior at high temperature and hence are expected to yield efficient TADF OLEDs. A sky blue emitter, **2AcNN**, showed a *η*_ext,max_ of 12% and a *η*_p,max_ of 28.8 lm W^−1^ with CIE (0.19, 0.36). Meanwhile, a green emitter, **2PXZNN**, realized a highly efficient TADF OLED realizing a *η*_ext,max_ of 20.8% with CIE (0.35, 0.55). Furthermore, by improving the carrier balance factor in the EML, the **2PXZNN**-based OLED achieved a low-operating voltage of 2.4 V at 1 cd m^−2^ and a high *η*_p,max_ of 71.6 lm W^−1^. We believe that these results clearly demonstrate the capability of pyridinecarbonitrile derivatives as TADF emitters, and contribute an effective molecular design for novel TADF emitters. Further study is ongoing in our laboratory.

## Experimental Section

### General Considerations

Quantum chemical calculations were performed using the Gaussian 09 program packages. An optimized structure was calculated at the B3LYP/6–31G(d) level for the ground state. The single-point energies were evaluated in accordance with the time-dependent density functional calculations in B3LYP/6-311 G+(d,p). ^1^H NMR and ^13^C NMR spectra were recorded on a JEOL 400 (400 MHz) spectrometer. Mass spectra were obtained using a Waters SQD2 mass spectrometer with an atmospheric pressure solid analysis probe (ASAP). DSC was performed using a Perkin-Elmer Diamond DSC Pyris instrument under a nitrogen atmosphere at a heating rate of 10°C min^−1^. TGA was undertaken using a SEIKO EXSTAR 6000 TG/DTA 6200 unit under a nitrogen atmosphere at a heating rate of 10°C min^−1^. UV–vis spectra was measured using a Shimadzu UV-3150 UV–vis–NIR spectrophotometer. Photoluminescence spectra were measured using a FluoroMax-2 (Jobin-Yvon-Spex) luminescence spectrometer. *I*_p_ was determined by a PYS under vacuum (=10^−3^ Pa). Transient PL decay curves and time-resolved photoluminescence spectra were measured using a streak camera (C4334 from Hamamatsu Photonics) at 5 and 300 K.

### Device Fabrication and Characterization

The substrates were cleaned with ultrapurified water and organic solvents (acetone, then isopropanol), and then dry-cleaned for 30 min through exposure to UV–ozone. Organic layers were deposited onto ITO substrates under vacuum (=10^−5^ Pa), successively. LiF and Al were patterned using a shadow mask with an array of 2 mm × 2 mm openings without breaking the vacuum (=10^−5^ Pa). The electroluminescent (EL) were taken using an optical multichannel analyzer Hamamatsu Photonics PMA-11. The current density–voltage and luminance–voltage characteristics were measured using a Keithley 2400 source measure unit and a Minolta CS200 luminance meter, respectively.

### Syntheses of Materials

#### Synthesis of 2AcNN

**AcPhBpin** (1.16 g, 2.8 mmol), 2-cloro-3-pyridinecarbonitrile (0.39 g, 2.8 mmol), and aqueous K_3_PO_4_ (1.35 M, 7.4 ml) were added to a round bottom flask. 1,4-Dioxane (35 ml) was added, and nitrogen bubbled through the mixture for an hour. Then, Pd_2_(dba)_3_ (0.13 g, 0.10 mmol) and S-phos (0.06 g, 0.10 mmol) were added, and the resultant mixture was stirred for 14 h at reflux temperature under N_2_ flow. The precipitate was filtered and washed with brine, dried over anhydrous MgSO_4_, filtered, and evaporated to dryness. The resulting solid was purified by chromatography on silica gel (eluent: toluene) to afford **2AcNN** (0.74 g, 67%) as a white solid: ^1^H NMR (400 MHz, DMSO-*d*_6_): δ = 9.01 (d, J = 5.0 Hz, 1H), 8.51 (d, J = 6.8 Hz, 1H), 8.20 (d, J = 8.2 Hz, 2H), 7.68 (dd, J = 7.9, 4.8 Hz, 1H), 7.59 (d, J = 8.2 Hz, 2H), 7.52 (d, J = 7.2 Hz, 2H), 7.02 (t, J = 7.5 Hz, 2H), 6.94 (t, J = 7.5 Hz, 2H), 6.23 (d, J = 8.2 Hz, 2H), 1.64 (s, 6H); ^13^C NMR (100 MHz, CDCl_3_): δ = 160.11, 152.82, 143.21, 142.04, 140.57, 136.91, 131.72, 131.49, 130.11, 126.44, 125.26, 121.95, 120.79, 117.54, 114.18, 107.56, 35.98, 31.20; MS: m/z = 388 [M+1]^+^; Anal calcd for C_27_H_21_N_3_: C, 83.69; H, 5.46; N, 10.84%. Found: C, 83.73; H, 5.38; N, 10.80%.

#### Synthesis of 2PXZNN

**PXZPhBpin** (1.28 g, 3.3 mmol), 2-cloro-3-pyridinecarbonitrile (0.48 g, 3.3 mmol), and aqueous K_3_PO_4_ (1.2 M, 8.7 ml) were added to a round bottom flask. 1,4-Dioxane (42 ml) was added, and nitrogen bubbled through the mixture for an hour. Then, Pd_2_(dba)_3_ (0.31 g, 0.33 mmol) and S-phos (0.14 g, 0.33 mmol) were added, and the resultant mixture was stirred for 18 h at reflux temperature under N_2_ flow. The precipitate was filtered and washed with brine, dried over anhydrous MgSO_4_, filtered, and evaporated to dryness. The resulting solid was purified by chromatography on silica gel (eluent: dichloromethane/hexane = 4:1) to afford **2PXZNN** (0.94 g, 78%) as an orange solid: ^1^H NMR (400 MHz, DMSO-*d*_6_): δ = 8.98 (dd, J = 4.8, 1.6 Hz, 1H), 8.49 (dd, J = 7.9, 1.6 Hz, 1H), 8.16 (d, J = 8.6 Hz, 2H), 7.62–7.68 (m, 3H), 6.67–6.78 (m, 6H), 5.91–5.94 (m, 2H) ppm; ^13^C NMR (100 MHz, CDCl_3_): δ = 159.93, 152.84, 143.88, 142.01, 140.90, 137.17, 133.92, 131.68, 131.26, 123.31, 122.03, 121.59, 117.48, 115.53, 113.37, 107.55; MS: m/z = 362 [M+1]^+^; Anal calcd for C_24_H_15_N_3_O: C, 79.76; H, 4.18; N, 11.63; O, 4.43%. Found: C, 79.73; H, 4.35; N, 11.54%.

#### Synthesis of 5PXZNN

**PXZPhBpin** (1.28 g, 3.3 mmol), 5-bromo-3-pyridinecarbonitrile (0.606 g, 3.3 mmol), and aqueous K_3_PO_4_ (1.2 M, 8.7 ml) were added to a round bottom flask. 1,4-Dioxane (42 ml) was added, and nitrogen bubbled through the mixture for an hour. Then, Pd_2_(dba)_3_ (0.31 g, 0.33 mmol) and S-phos (0.14 g, 0.33 mmol) were added, and the resultant mixture was stirred for 17 h at reflux temperature under N_2_ flow. The precipitate was filtered and washed with brine, dried over anhydrous MgSO_4_, filtered, and evaporated to dryness. The resulting solid was purified by chromatography on silica gel (eluent: dichloromethane) to afford **5PXZNN** (0.91 g, 76%) as a yellow solid: ^1^H NMR (400 MHz, DMSO-*d*_6_): δ = 9.31 (d, J = 2.3 Hz, 1H), 9.07 (d, J = 1.4 Hz, 1H), 8.77 (t, J = 2.0 Hz, 1H), 8.13 (d, J = 8.6 Hz, 2H), 7.60 (d, J = 8.6 Hz, 2H), 6.65–6.77 (m, 6H), 5.91–5.93 (m, 2H) ppm; ^13^C NMR (100 MHz, CDCl_3_): δ = 151.48, 151.12, 143.91, 140.22, 137.34, 136.00, 135.56, 133.93, 132.12, 129.89, 123.27, 121.71, 116.41, 115.65, 113.16, 110.26; MS: m/z = 362 [M+1]^+^; Anal calcd for C_24_H_15_N_3_O: C, 79.76; H, 4.18; N, 11.63; O, 4.43%. Found: C, 79.71; H, 4.04; N, 11.63%.

## Author Contributions

HS conceived the project. HS and YM interpreted the data. HS and JK supervised the project. HS, NO, and YM designed the experiments. YM, HA, and NO prepared the samples and performed the data analyses. YM and HA synthesized and characterized the materials. YM and HA performed the quantum chemical calculations. YM and HS prepared the manuscript and supplementary materials. All authors discussed the results and commented on the manuscript.

### Conflict of Interest Statement

The authors declare that the research was conducted in the absence of any commercial or financial relationships that could be construed as a potential conflict of interest.
