# Supplementary material for: High-Efficiency Sky Blue-To-Green Fluorescent Emitters Based on 3-Pyridinecarbonitrile Derivatives
Source: Front Chem. 2019 Apr 24;7:254. doi: 10.3389/fchem.2019.00254 (PMC6491696; doi:10.3389/fchem.2019.00254)
Supplement: Supplementary file 1 [file Data_Sheet_1.PDF]

Supplementary Material **for:**

High Efficiency Sky Blue-to-Green Fluorescent Emitters based on 3-Pyridinecarbonitrile Derivatives

Yuki Masuda[1], Hisahiro Sasabe[1,2,3]*, Hiroki Arai[1], Natsuki Onuma[1], Junji Kido[1,2,3]*

[1]Department of Organic Materials Science, Graduate School of Organic Materials Science, Yamagata University, 4-3-16 Jonan, Yonezawa, Yamagata 992-8510, Japan

[2]Frontier Center for Organic Materials (FROM) Yamagata University

[3]Research Center for Organic Electronics (ROEL) Yamagata University

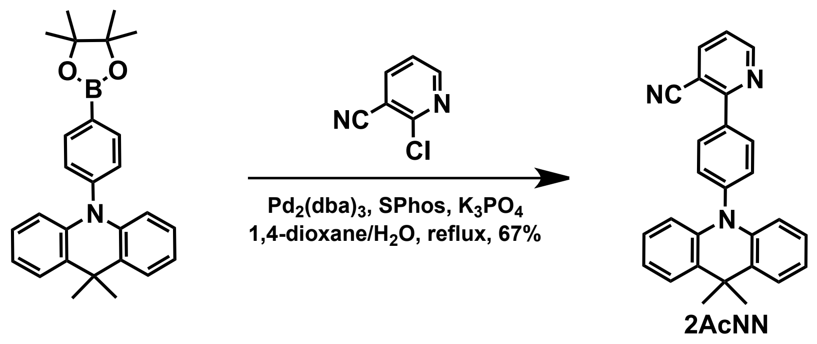

Scheme S1. Synthetic routes of **2AcNN**.

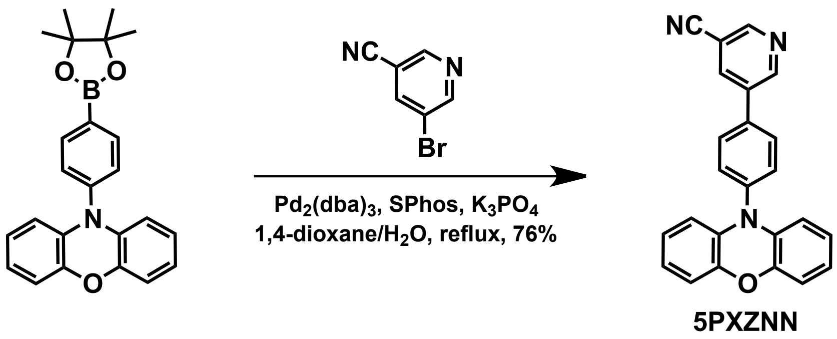

Scheme S2. Synthetic routes of **5PXZNN**.

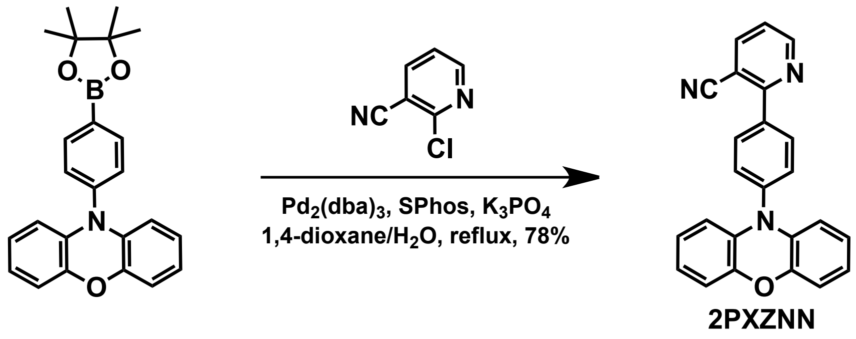

Scheme S3. Synthetic routes of 2**PXZNN**.

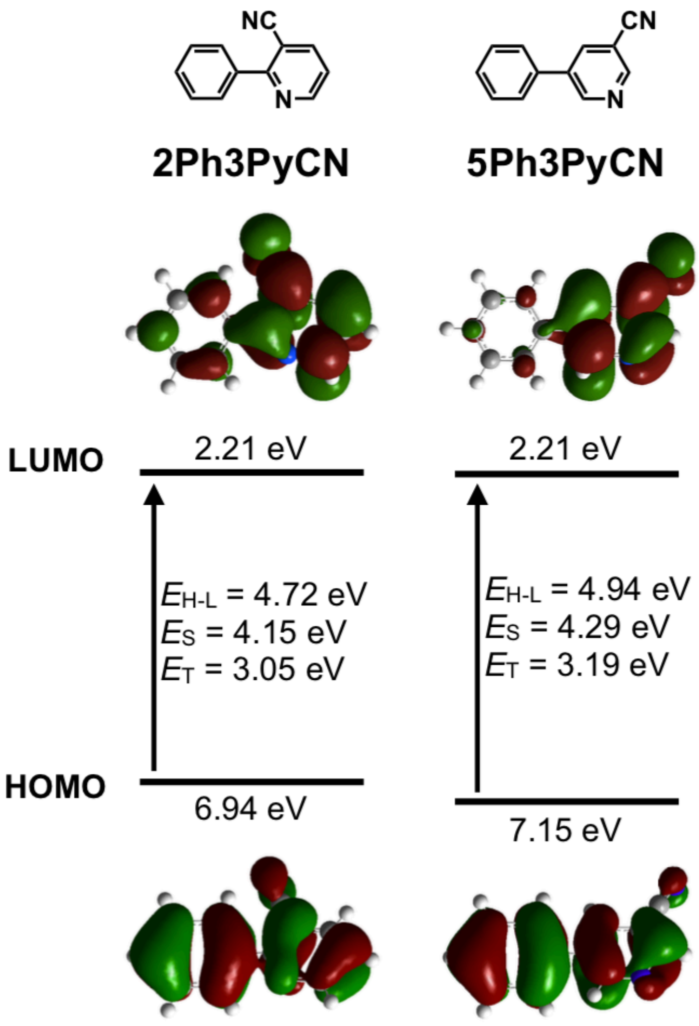

Figure S1. DFT calculations of phenyl-3-pyridinecarbonitriles.

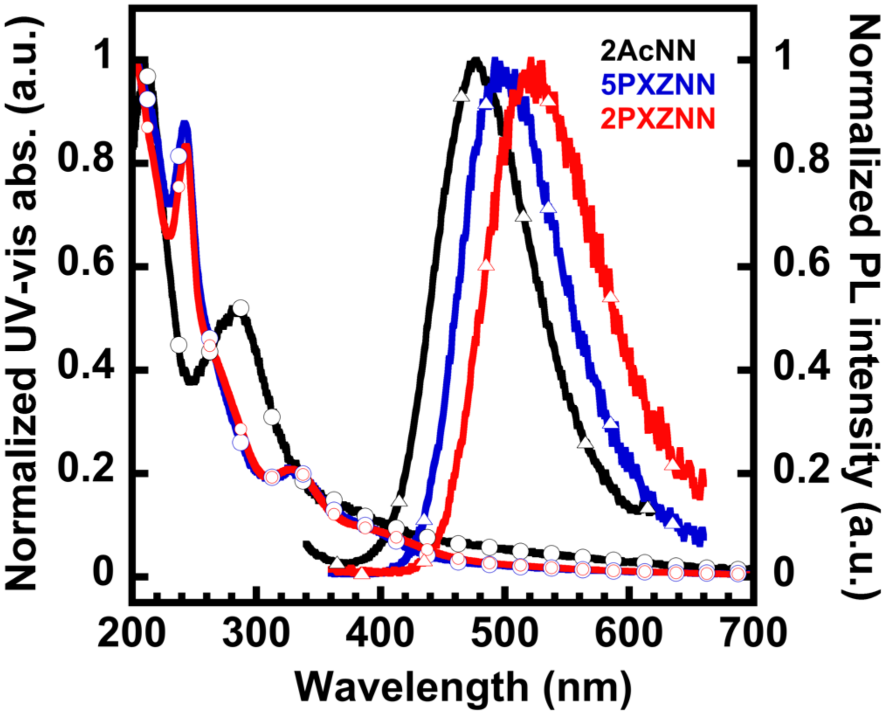

Figure S2. UV-vis absorption spectra of **2AcNN**, **5PXZNN**, and **2PXZNN** neat film. PL spectra of 10wt% 2AcNN-, 5PXZNN-, and 2PXZNN-doped DPEPO films.

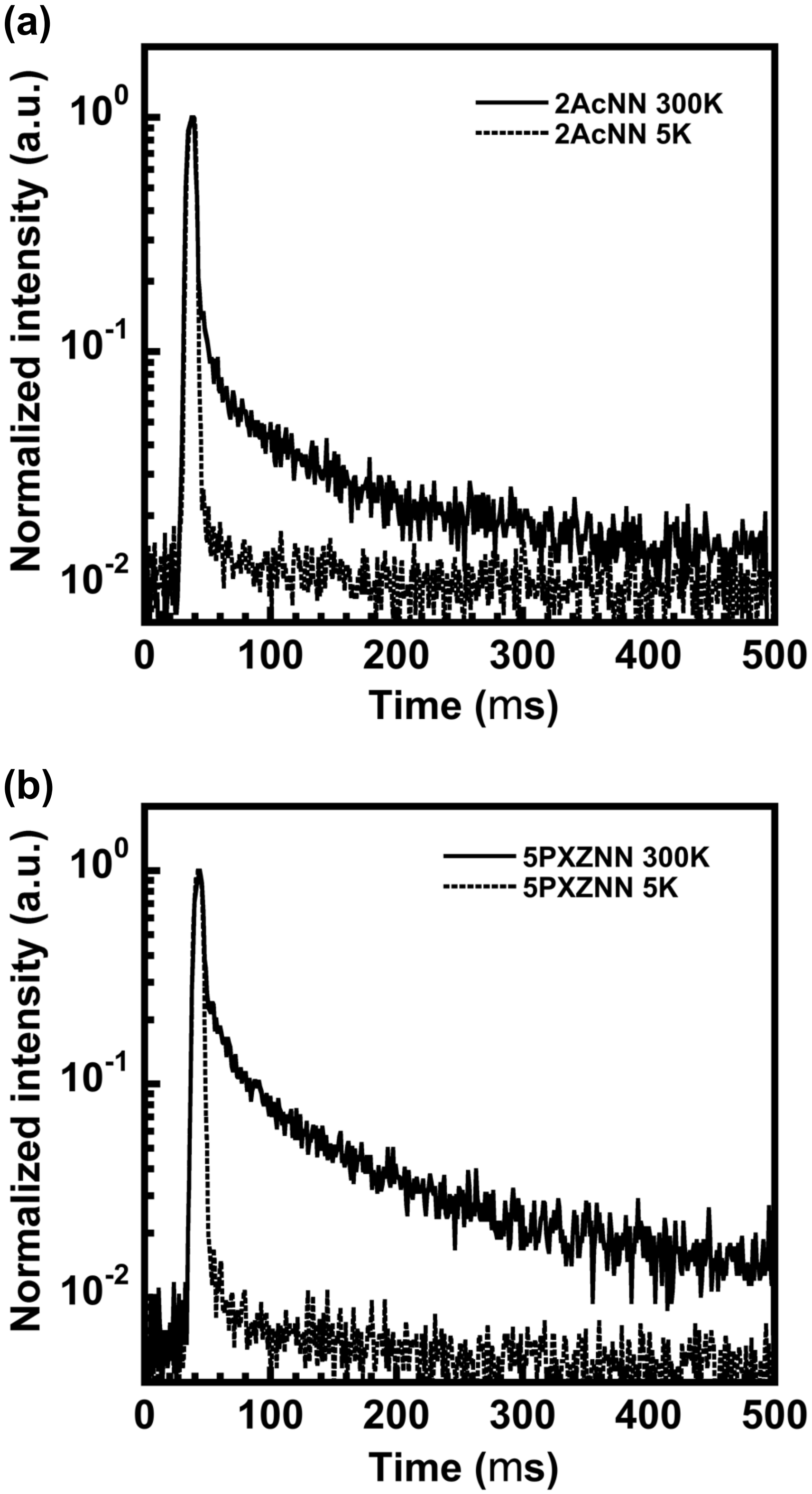

Figure S3. Transient photoluminescent decay curves at 5 K and 300 K of (a) **2AcNN**, and (b) **5PXZNN**.

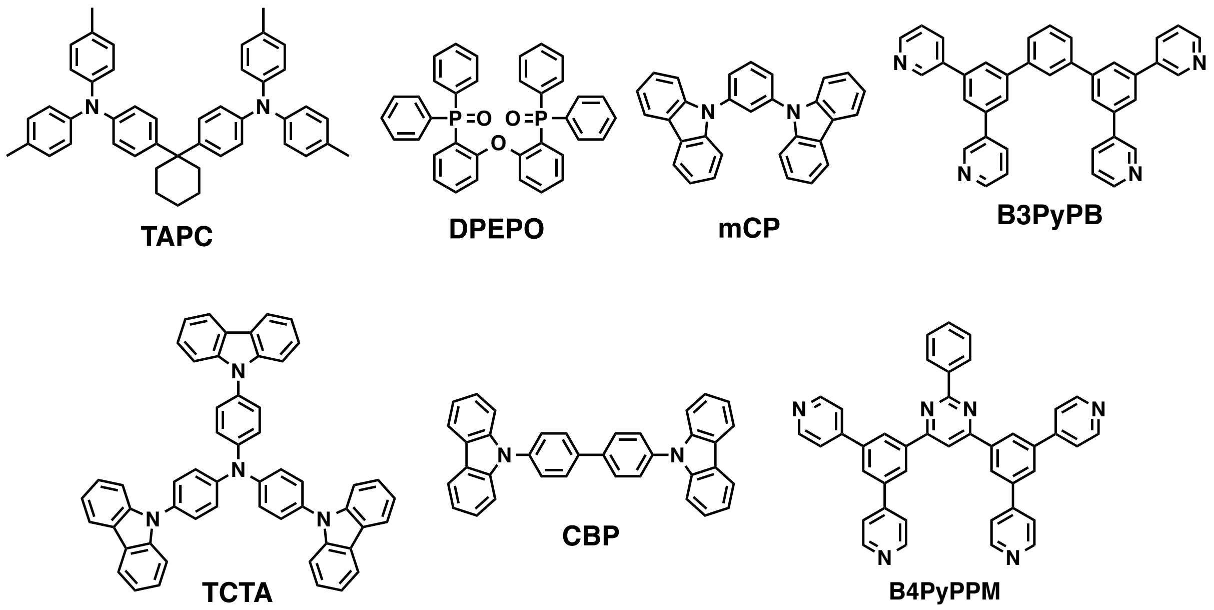

Figure S4. Chemical structures of materials used in this study.

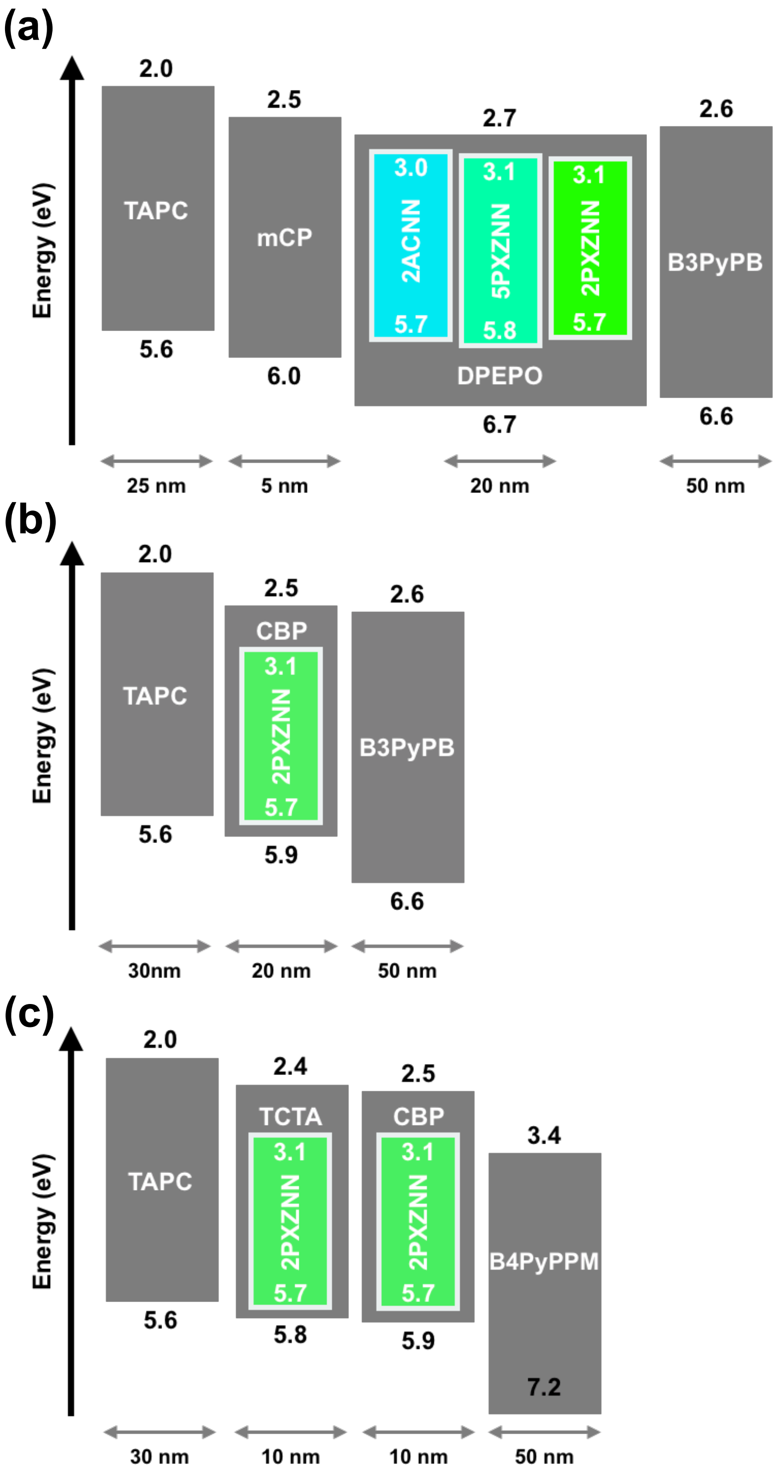

Figure S5. Energy diagrams of OLEDs in this study.

2. Supplementary Tables

Table S1. DFT calculation results of **2AcNN**, **5PXZNN**, and **2PXZNN**

Compounds	HOMO [eV]	LUMO [eV]	$\Delta E_{H\text{-}L}$ [eV]	E_S [eV]	E_T [eV]	ΔE_{ST} [eV]	f
**2AcNN**	−5.24	−2.37	2.86	2.39	2.38	0.006	0.0002
**2PXZNN**	−5.04	−2.43	2.61	2.15	2.13	0.023	0.0131
**5PXZNN**	−5.22	−2.39	2.83	2.40	2.39	0.006	0.0013

Table S2. Optical properties of **2AcNN**, **5PXZNN**, and **2PXZNN**

Compounds	λ_{abs}[a)] [nm]	λ_{PL} [nm]		
		sol.[a)]	neat film	doped film
**2AcNN**	364	492	503	477[b)]
**2PXZNN**	390	550	551	521[b)]/522[c)]
**5PXZNN**	375	503	519	492[b)]/492[c)]

a) Measured in toluene solution, b) measured in 10wt%-emitter doped DPEPO films, c) CBP was used as a host material instead of DPEPO.
